# Molecular profiling for personalized cancer care

**DOI:** 10.1007/s10585-012-9483-3

**Published:** 2012-06-08

**Authors:** Raheela Ashfaq

**Affiliations:** Caris Life Sciences, 6655 North MacArthur Blvd., 3rd Floor, Irving, TX 75039 USA

**Keywords:** Molecular profiling, Targets, Targeted therapies, Molecular diagnostics, Clinical trials

## Abstract

The pace of genomic discoveries in the field of cancer is revolutionizing our understanding of the biological dynamics of cancerous growth and, at the same time, fueling research for newer and smarter cancer therapies to reverse the effects of these alterations. These dynamics are driving a tremendous paradigm shift in cancer diagnostics, drug development and clinical trial design with the hope of eliminating the current structure and approach of cancer care, to one which is driven by the underlying biology of the tumor and, thus highly personalized. Much of this paradigm shift has been fueled by the current availability of novel technologies, platforms and bioinformatic tools. Today, therapies are being rationally designed to target the precise genetic alterations with better clinical outcomes with reduced morbidity. Therefore, molecular profiling of tumors to identify the multiplicity of alterations in a tumor is an essential and necessary companion for targeted therapies.

## Introduction

Molecular profiling (MP) encompasses the testing of multiple biomarkers to evaluate the underlying genetic alterations present in a tumor at any one point in time [1–3]. To date molecular profiles may consist of multiple gene mutational analyses, gene copy number changes by fluorescence in situ hybridization (FISH), gene expression profiles measured by microarrays (MA) and protein expression by immunohistochemistry (IHC). This approach is superior to the testing of one biomarker target which does not take into account the complexity of multiple signaling pathways and cross talk [4].

The cancer literature is replete with studies exploring single biomarkers in clinical trials designed to test single agents or biomarker analyses performed as a post hoc analysis. Although there are a plethora of biomarkers that have emerged that may positively prognosticate or predict response to various therapies, the clinical utility and adoption of this approach has been slow due to validation concerns, reproducibility and translation into clinical care [5–7].

Since comprehensive MP uses a multi-dimensional approach to testing, it is inherently more complex and requires extensive validation. There is significant investment in high cost, high throughput technologies, trained laboratory work force, and laboratory informatics to achieve the level of validation required by CLIA or CAP to offer the test for patient-care. Laboratory developed test-validations in a CLIA mandated environment typically requires the following [8]:

- Specimen type and specimen handling protocols: since this variable can hugely affect reproducibility of the test, standardization of specimen handling is imperative. More and more, the formalin fixed paraffin embedded tissue is becoming the sample of choice as it is readily available. This sample type has been shown to perform adequately for mutational analysis; gene expression profiles measured by RT-PCR or oligonucleotide arrays, FISH, and IHC. However, the quality of analysis on this preferred sample type has to be closely monitored based on time to fixation, time in fixative and age of the samples. Additionally for newer molecular techniques, internal and external quality checks such as the amount or percentage of tumor nuclei, the quantity and quality of DNA and RNA, measurement of internal housekeeping genes are all important determinants in the overall quality of results [9–15]. Validation of each component of the MP assay, whether being performed in a non-profit hospital-based/academic laboratory or for-profit reference labs, has to follow the strict CLIA guidelines as well as guidelines provided by laboratory associations such as College of American Pathologists (CAP) or CLS1. For each test offered, per validation guidelines, the laboratory must document certain performance characteristics which include:
  - Accuracy to document that the test produces expected result by appropriate testing of known positive and negative samples. From these accuracy studies the analytical sensitivity, specificity and accuracy of the assay can be determined
  - Precision studies are performed to determine intra-run and inter-run reproducibility.
  - The assay will also have to determine appropriate reference ranges and limit of detection for appropriate reporting of results. Ongoing quality assurance and proficiency testing are some other additional requirements by CLIA

Given the resource investment requirements for conducting these multi-dimensional, labor intense assays, it is easily conceivable that these assays are increasingly being offered by large centralized laboratories. (examples include: Genomic Health Inc., Pathwork Diagnostics and Caris Life Sciences). The rapidly developing genomic information is leading to the proliferation of MP services and assays and their subsequent introduction into clinical care.

One such MP service is the Caris Target Now^™^. This service offers a new approach in which an evidence rated review of the literature based on the the US Preventive Task Force rating is utilized to identify targets in tumor tissue associated with current therapies [16]. Using a technology and platform agnostic approach, various targets are analyzed using a combination of assays such as gene sequencing, oligonucleotide microarray, mutational analyses, copy number changes using FISH analysis and protein expression by IHC.

This particular approach for MP to measure molecular targets was studied in a feasibility study in 2006 [17] and most recently in a multi-center clinical trial, across nine different cancer centers in the US. Using the Caris Life Sciences Caris Target Now^™^ MP service, Von Hoff et al. [18] reported a longer PFS for patients on MP-directed therapy than physician choice for 27 % of patients (95 % CI, 17–38 % *P* = 0.007). This study used a novel study design in which the patient served as their own controls and PFS ratio was determined by actual comparison of PFS on MP therapy versus PFS on patient’s last prior therapy. For the participants (18/66) who had a PFS ≥1.3 overall survival was 9.7 months compared to 5 months on physician directed therapy. Interestingly, MP of tumors yielded actionable targets in 98 % by this assay indicating that such an approach is feasible. However, it is to be noted that the targets identified may involve off-label use of therapies [18]. Whereas Von Hoff et al. study was restricted to advanced stage patients with metastases and refractory tumors, the approach may have significant benefits when used earlier.

Using the same Caris Target Now^™^ service, Shacham-Shmuel et al. [19], reported two patients with advanced stage colon cancers in which identification of a target MGMT by IHC with this assay, led to measurable response to temozolomide treatment with decrease in serum markers and tumor shrinkage on CT. Using the same assay, discovery of targets was also reported in a interdigitating reticulum cell sarcoma, an exceedingly rare tumor [20]. Tsimberdou et al. [21] presented the MD Anderson experience using MP. Median time to treatment failure (TTF) in 161 patients with one aberration treated with matched targeted therapy was 5.3 months (95 % CI: 4.1, 6.6) versus 3.2 months (95 % CI: 2.9–4.0) for their prior systemic antitumor therapy (prior to referral to phase I) (*P* = 0.0003). For patients with one aberration, the CR-PR rate was 29 % with matched targeted therapy versus 8 % without matching (*P* = 0.0001). The CR + PR rate was 6 % in 438 patients without molecular testing treated on the same studies. Interestingly, these rates compare favorably with those reported by Von Hoff et al. for the Caris Target Now service. These preliminary results suggest that in early clinical trials, matching patients with targeted drugs based on their molecular profile results in (a) longer TTF compared to their prior therapy and (b) higher rates of response, survival and TTF compared to those seen in patients treated without molecular matching. The Battle trial for personalizing therapy for lung cancer identified targets of high interest in treatment of lung cancer and using an adaptive randomized trial utilized real time biomarker analyses to predict sensitivity or resistance to targeted agents [22]. A similar trial I-Spy 2 also employs this groundbreaking clinical trial model that uses genetic or biological markers (“biomarkers”) from individual patient’s tumors to screen promising new treatments, identifying which treatments are most effective in specific types of patients [23]. In addition, this innovative adaptive trial design similar to Battle Trial will enable researchers to use early data from one set of patients to guide decisions about which treatments might be more useful for patients later in the trial, and eliminate ineffective treatments more quickly.

With more focus on the MP of tumors and greater realization of the limitations of one-biomarker—one target approach, cancer treatment in the US is about to experience a major revolution. Upfront MP of tumors at the time of diagnosis and subsequently at all points of tumor recurrence, whether local or distant, will change the treatment of oncology care forever. This will hopefully lead to better control of cancer, improved outcomes for patients, and a more rational and less expensive oncology care.
